# Community-Based Participatory Research on Diet and Activity With an Indigenous Pueblo Community

**DOI:** 10.1093/geroni/igab046.898

**Published:** 2021-12-17

**Authors:** Karen Kopera-Frye

**Affiliations:** New Mexico State University, Las Cruces, New Mexico, United States

## Abstract

Tribal Critical Race Theory (Brayboy, 2005) supports the use of decolonizing methodologies such as Community-Based Participatory Research when collaborating with Indigenous communities. This paper highlights the underlying processes in working with a Pueblo community on an intergenerational health project. Indigenous participants included 16 Piro Pueblo individuals who collaborated on a project examining healthy diets and activity in their community. The project involved providing information on the importance of activity and healthy eating of traditional foods to promote healthy living. Thematic analysis of open-ended questions exploring the role of culture in food and activity yielded important themes of gathering, resilience, history, honoring ancestors at mealtime, cultural ways, and activities such as dancing and drumming. The results suggest that projects addressing diet and activity collaborate to ensure cultural values, e.g., connectedness, cultural ways, e.g., gathering, dancing, and Indigenous knowledge are represented in the project and viewed through an Indigenous lens.

